# Economic Costs of Childhood Lead Exposure in Low- and Middle-Income Countries

**DOI:** 10.1289/ehp.1206424

**Published:** 2013-06-25

**Authors:** Teresa M. Attina, Leonardo Trasande

**Affiliations:** 1 Department of Pediatrics,; 2 Department of Environmental Medicine, and; 3 Department of Population Health, New York University School of Medicine, New York, New York, USA; 4 Wagner School of Public Service, New York University, New York, New York, USA; 5 Department of Nutrition, Food Studies, and Public Health, Steinhardt School of Culture, Education, and Human Development, New York University, New York, New York, USA

## Abstract

Background: Children’s blood lead levels have declined worldwide, especially after the removal of lead in gasoline. However, significant exposure remains, particularly in low- and middle-income countries. To date, there have been no global estimates of the costs related to lead exposure in children in developing countries.

Objective: Our main aim was to estimate the economic costs attributable to childhood lead exposure in low- and middle-income countries.

Methods: We developed a regression model to estimate mean blood lead levels in our population of interest, represented by each 1-year cohort of children < 5 years of age. We used an environmentally attributable fraction model to estimate lead-attributable economic costs and limited our analysis to the neurodevelopmental impacts of lead, assessed as decrements in IQ points. Our main outcome was lost lifetime economic productivity due to early childhood exposure.

Results: We estimated a total cost of $977 billions of international dollars in low- and middle-income countries, with economic losses equal to $134.7 billion in Africa [4.03% of gross domestic product (GDP)], $142.3 billion in Latin America and the Caribbean (2.04% of GDP), and $699.9 billion in Asia (1.88% of GDP). Our sensitivity analysis indicates a total economic loss in the range of $728.6–1162.5 billion.

Conclusions: We estimated that, in low- and middle-income countries, the burden associated with childhood lead exposure amounts to 1.20% of world GDP in 2011. For comparison, in the United States and Europe lead-attributable economic costs have been estimated at $50.9 and $55 billion, respectively, suggesting that the largest burden of lead exposure is now borne by low- and middle-income countries.

Citation: Attina TM, Trasande L. 2013. Economic costs of childhood lead exposure in low- and middle-income countries. Environ Health Perspect 121:1097–1102; http://dx.doi.org/10.1289/ehp.1206424

## Introduction

The removal of lead from gasoline is perhaps one of the greatest public health accomplishments, and arguably produced some of the largest reductions in pediatric morbidity, over the past 50 years. As a result of an aggressive international campaign by the United Nations Environment Programme (UNEP), today only six countries continue to use leaded gasoline (UNEP 2012). Before the removal, especially in urban areas, children inhaled or ingested lead liberated as a result of the combustion of leaded gasoline, leading to large-scale increases in blood lead levels (BLLs) and associated adverse health consequences, including cognitive and behavioral deficits (Agency for Toxic Substances and Disease Registry 2007). The average child’s BLL has decreased substantially: In the 1970s, > 88% of children 1–5 years of age in the United States had BLL ≥ 10 μg/dL, whereas the most recent data, collected in 2007–2008, show average levels of 1.5 μg/dL, with only 0.9% of children having BLL > 10 μg/dL [U.S. Environmental Protection Agency (EPA) 2012]. A similar trend has also been documented in most European countries (UNEP 2010), as well as in some low- and middle-income countries (LMICs) (Norman et al. 2007).

Yet despite this major landmark accomplishment, significant exposure remains, especially in LMICs (Fewtrell et al. 2004). Lead consumption has significantly increased since 1970 (from 4.7 million to ~ 7.1 million tons in 2004), an increase driven mainly by demand for lead batteries (UNEP 2010). Paint is still a major source of lead exposure in childhood: Lead paint is used globally to this day, resulting in contaminated dust in homes, which is then either ingested or inhaled (UNEP 2010). Hazardous waste sites also represent a major source of contamination of water, soil, and food, leading to increases in BLL in children from surrounding communities (UNEP 2012). Other environmental sources include water pipes, solder in canned food, ceramics, and traditional remedies (UNEP 2010).

A growing body of literature in recent years has estimated disability-adjusted life year (DALY) losses from exposure to lead in children at the global level (Fewtrell et al. 2004; Murray et al. 2012). Although DALYs are highly useful for prioritizing public health interventions in general, for environmental health interventions cost estimates represent a complementary assessment of burden that can be compared directly with costs of reducing exposure. Although costs of childhood lead exposure in the United States (Gould 2009; Trasande and Liu 2011) have proven useful for decision makers there, to date there have been no estimates of costs related to childhood lead exposure in developing countries.

Here we estimate the economic costs attributable to childhood lead exposure in low- and middle-income countries (Table 1).

**Table 1:** Countries included in the study by WHO region.

WHO region	Country
Africa
Eastern Africa	Burundi, Comoros, Djibouti, Eritrea, Ethiopia, Kenya, Madagascar, Malawi, Mauritius, Mozambique, Rwanda, Somalia, Uganda, Tanzania, Zambia, Zimbabwe
Southern Africa	Botswana, Lesotho, Namibia, South Africa, Swaziland
Western Africa	Benin, Burkina Faso, Cape Verde, Cote d’Ivoire, Gambia, Ghana, Guinea, Guinea Bissau, Liberia, Mali, Mauritania, Niger, Nigeria, Senegal, Sierra Leone, Togo
Middle Africa	Angola, Cameroon, Central African Republic, Chad, Congo, Democratic Republic of Congo, São Tomé and Principe
Northern Africa	Algeria, Egypt, Libya, Morocco, Sudan, Tunisia
Asia
Eastern Asia	Democratic People’s Republic of China, Mongolia
Southern Asia	Afghanistan, Bangladesh, Bhutan, India, Iran, Nepal, Pakistan, Sri Lanka
Central Asia	Kazakhstan, Kyrgyzstan, Tajikistan, Turkmenistan, Uzbekistan
Southeastern Asia	Cambodia, Indonesia, Lao People’s Democratic Republic, Malaysia, Myanmar, Philippines, Thailand, Timor Leste, Vietnam
Western Asia	Armenia, Azerbaijan, Georgia, Iraq, Jordan, Lebanon, Syria, Turkey, Yemen
Latin America/Caribbean
Central America	Belize, Costa Rica, El Salvador, Guatemala, Honduras, Mexico, Nicaragua, Panama
South America	Argentina, Bolivia, Brazil, Chile, Colombia, Ecuador, Guyana, Paraguay, Peru, Suriname, Uruguay, Venezuela
Caribbean	Dominican Republic, Grenada, Haiti, Jamaica, Saint Lucia, Saint Vincent and the Grenadines
WHO, World Health Organization. Countries not included in the economic analysis because either no GDP per capita data or no data on population <5 years were available: Seychelles, North Korea, Occupied Palestinian Territory, Antigua and Barbuda, Cuba, Dominica, and Saint Kitts and Nevis. Oceania is not included because the vast majority of the population reside in Australia and New Zealand, both high-income countries.	

## Methods

*General description*. We applied the model first used by the Institute of Medicine (1981) to estimate the cost of environmentally mediated disease.

Although BLLs reflect mainly the exposure to lead that occurred in the previous few months, and may not reflect the burden of lead in bones (Needleman et al. 1996), BLL is the most commonly available measure. Increases in BLL among children are associated with decrements in cognitive development, as quantified in IQ loss. We limited our economic analysis to the neurodevelopmental impact of lead, assessed as decrements in IQ point loss estimated over three ranges of BLLs: 0.513 IQ point loss per 1-μg/dL for BLL 2–10 μg/dL; 0.19 point loss for BLL 10–20 μg/dL; and 0.11 point loss for BLL ≥ 20 μg/dL, as described by Gould (2009). We focused on the population at risk represented by each 1-year cohort of children < 5 years of age, in whom the BLL, when measured longitudinally, is most strongly associated with neurodevelopment at school age (Hornung et al. 2009).

We did not include mild mental retardation (MMR) in our cost estimates, because cost estimates for MMR are rarely available outside the developed world. Decrements in IQ are associated with reduced lifetime economic productivity, and associations with criminality have also been identified (Needleman et al. 1996; Reyes 2007), but these to date are limited to industrialized countries. Therefore, we did not include costs of increased criminality in our estimates of economic costs to LMICs.

*Environmentally attributable fraction*. We applied an environmentally attributable fraction (EAF) (Smith et al. 1999) of 100%, consistent with scientific literature indicating that only a very small fraction of lead exposure is attributable to natural processes (UNEP 2010). Accordingly, the attributable cost can be described as follows:

Cost = EAF × BLL × (IQ loss/BLL) × (lost economic productivity/IQ loss) × population at risk. [1]

In this equation, IQ is the IQ loss for each BLL range of values, as described above, and the population at risk is represented by each 1-year cohort of children < 5 years of age. We estimated the number in each cohort as 20% of the total number of 0- to 4-year-old children reported for each country by the most recent UN estimates (United Nations 2012).

*Estimation of BLL distributions at the country level*. We systematically reviewed the published literature for studies estimating BLLs in LMICs, following the most recent World Bank country classification by income (World Bank 2012a). The published literature was searched using PubMed (http://www.ncbi.nlm.nih.gov/entrez/) and terms including “lead” in combination with the name of each country; the initial query was then refined using the “Related Citations” option. We also considered reference lists of relevant articles. We included only studies conducted from 2000 onward (or for which the recruitment period extended to the year 2000), in pediatric populations (< 18 years of age) or that included a pediatric subpopulation. Studies reporting lead exposure in heavily contaminated areas (hot spots such as areas around metal smelters and battery-recycling or gold ore–processing activities—the latter responsible for the recent outbreak of fatal lead poisoning in children in Nigeria (Dooyema et al. 2012)—or occupational exposures were excluded, unless they included a control population not residing in the contaminated area. In these latter cases, we analyzed only data from the control population. Country-specific BLL estimates identified based on our review and used in the present analysis are provided along with the sources of these data in Supplemental Material, Table S1.

For this analysis, we did not consider urban and rural populations separately, because there is a global trend toward urbanization, and more than half of the world’s population now lives in urbanized areas, with urban growth concentrated in Africa and Asia (United Nations 2011).

*Estimating BLL from past studies.* We developed a regression model to relate trends in BLLs over time, and to relate these to the timing of the ban in leaded gasoline in each country; this was done using BLL data retrieved through our literature search. We first examined a simple linear model with respect to trends in BLL over time, and compared our results with a linear plus quadratic model, which resulted in a modest increase of the predictive capability, measured using the *R*^2^ coefficient of determination. Therefore, our final model included a quadratic term, and is described by the following regression equation:

*y*(*t*) *=* β_0_ *–* β_1_*x +* β_2_*x*^2^ *+ e*, [2]

where *y* is the average BLL at time *t* (2008), β_0_ is the intercept, *x* is the difference between year of the study and year of leaded gasoline phaseout in the country (UNEP 2012), *x*^2^ is the quadratic term of the difference, and β_1_ and β_2_ are the coefficients being estimated. The quadratic term is also justified by experience in developed countries, in which the most rapid reductions in childhood blood levels were produced immediately after phaseout and in relationship to more rapid reductions in leaded gasoline use (Fewtrell et al. 2004; U.S. EPA 2003).

Parameter estimates obtained with this model are shown in Table 2, and were used to estimate BLL in each of the countries included in our analysis. Below is a working example for a specific country, Ethiopia, which has no recent BLL data available and in which leaded gasoline was phased out in 2004:

**Table 2:** Model parameter estimates for BLLs and related SDs predicted for each country in 2008.

Model parameter	Mean (95% CI)	*p*-Value
BLL
Intercept (β_0_), BLL (μg/dL)^*a*^	7.33 (6.18, 8.48)	<0.001
Time coefficient (*x*)	–0.26 (–0.43, –0.09)	0.003
Quadratic time coefficient (*x*^2^)	0.01 (–0.01, 0.04)	0.31
*R*^2^	0.12	
SD
Intercept (β_0_), SD (μg/dL)^*a*^	0.27 (–0.86, 1.41)	0.63
BLL coefficient (*x*_1_)	0.47 (0.33, 0.60)	<0.001
Time coefficient (*x*_2_)	0.14 (0.05, 0.24)	0.005
Quadratic time coefficient (*x*_2_^2^)	–0.001 (–0.023, 0.01)	0.89
*R*^2^	0.54
^***a***^For countries where BLL and SD data were available, the country’s actual value was used.		

BLL in 2008 = [7.33 – (0.26 ×⊇4) + (0.01 × 16)] = 6.45 μg/dL.

We used the same model to derive SD values, but with the inclusion of BLL as one of the coefficients:

*y*(*t*) *=* β_0_ *+* β_1_*x*_1_ + β_2_*x*_2_ – β_3_*x*_2_^2^ *+ e_i_*, [3]

where *y* is the average SD at time *t* (2008), β_0_ is the intercept, *x*_1_ is the average BLL, *x*_2_ is the difference between year of the study and year of leaded gasoline phaseout, *x*_2_^2^ is the quadratic term of the difference, and β_1_, β_2_, and β_3_ are the coefficients being estimated. Therefore, for Ethiopia, we estimated the following SD:

SD in 2008 = [0.27 + (0.47 × 6.45) + (0.14 × 4) – (0.001 × 16)] = 3.85 μg/dL.

For countries with available data, the actual BLL and SD values were used in the regression equation; if the data were collected after 2008, we subtracted 2008 from the year of the study and used the difference. For some of these countries, more than one study reporting blood lead concentrations was available. In this case, we first estimated BLL levels in 2008 using our regression model and then combined these estimates to derive a single, sample size–weighted, geometric mean, according to a method previously described by Fewtrell et al. (2003). An example of this is provided in the Supplemental Material, Methods.

The same procedure was followed to combine SDs. Once we estimated mean BLL and SD for each country, the percentage of children at or above predefined blood levels intervals (2–10, 11–19, ≥ 20) was estimated to determine the population at risk within each exposure interval assuming a log-normal distribution around the estimated mean BLL using the LOGNORMDIST function in Excel 2007 (Microsoft, Inc., Redmond, WA). For this study, we considered BLL < 2 µg/dL to present the lowest risk of toxic effects in children, acknowledging that a threshold level does not appear to exist.

*IQ loss.* Current evidence supports impaired cognitive development associated with lead concentrations < 10 µg/dL, and a nonlinear, inverse relationship between IQ and BLL has been established (with the greatest rate of IQ loss per unit blood lead < 10 µg/dL). Average IQ point loss was derived from an international pooled analysis (Lanphear et al. 2005), over three ranges (0.513 point loss per 1-μg/dL for BLL 2–10 μg/dL; 0.19 point loss for BLL 10–20 μg/dL; and 0.11 point loss for BLL ≥ 20 μg/dL), as described by Gould (2009). Because of the broad range of BLL ≥ 20 μg/dL, we also divided the ≥ 20 μg/dL group into 20–44, 45–69, and ≥ 70 μg/dL for analysis. For each of these BLL ranges, we applied the IQ point loss corresponding to the lowest BLL in the range considered (e.g., for the range 2–10 μg/dL, we applied the IQ loss corresponding to 2 μg/dL). IQ loss was calculated for each country using the BLL estimated for that country multiplied by the number of children < 5 years of age affected each year. IQ losses for each country were then summed to obtain a total for each subregion in Africa, Asia, and Latin America/Caribbean.

*Losses in economic productivity.* To estimate lead-attributable costs, the economic model developed by Schwartz et al. (1985) was applied to the calculated prevalence distribution. This model is based on the relationship between lead exposure and dose-related decrements in IQ score, the latter in turn being associated with decreased lifetime earning power.

We estimated lost lifetime economic productivity (LEP) using average IQ point loss per microgram per deciliter BLL, percent lost LEP per IQ point, and total lost LEP. Lost LEP was derived based on a U.S. estimate (Grosse et al. 2002) of decrements in LEP per IQ point loss. For our base-case analysis, we assumed a 2% loss in LEP–IQ point estimate, as previously done (Trasande and Liu 2011), against LEP data from the University of California Institute for Health and Aging, which assume annual growth in productivity of 1% and a 3% discount rate (Max et al. 2007). These data suggest that the value of lifetime expected earnings is $1,413,313 for a 5-year-old boy in 2007 and $1,156,157 for a 5-year-old girl. These data were then corrected at the country level using gross domestic product (GDP) by converting GDP per capita to international dollars using purchasing power parity (PPP) rates (World Bank 2012b). All monetary amounts reported are in international dollars, unless otherwise specified.

*Sensitivity analyses.* Recognizing uncertainty in LEP–IQ and in trends in BLL, we performed two types of sensitivity analysis to increase the accuracy of our estimates. First, we applied the method used by Fewtrell et al. (2004) to estimate the exposure distributions in our population of interest. Following this approach, we also accounted for lead-reduction programs that were undertaken after BLLs were surveyed. We used a reduction factor of 7.8% decrease per year (Fewtrell et al. 2004), taking into account the year of the study and the year of leaded gasoline phaseout in each country with available data. For countries with more than one study reporting BLL, we derived a single, sample size–weighted, geometric mean BLL value and SD (for more details, see Supplemental Material, Methods.) We then obtained a subregional mean BLL by weighting country means by the size of the population < 5 years of age. For countries for which BLL data were not available, we used the corresponding subregional mean and SD to estimate the population distribution of exposure, assuming a log-normal distribution around the mean BLL for the subregion. Unlike our regression model, this method does not allow for an estimation of BLL at country level for those countries with no recent data available, and uses instead the corresponding subregional BLL mean as a substitute.

Second, recognizing the uncertainty in the relationship between IQ and economic productivity, we used the low and high ends of our estimate range based on the work of Schwartz et al. (1985) and Salkever (1995), who applied a range in percentages of lifetime productivity loss for each point of IQ ranging from 1.76% to 2.37%.

## Results

*Regression model for BLL.* Model parameter estimates are presented in Table 2. The model predicted a significant inverse relationship between BLL and time, represented by the difference between year of the study and year of leaded gasoline phaseout. Estimates show a significant positive relationship of SD with BLL, with wider dispersion over time.

*Base-case analysis.* Results are presented for Africa, Asia, and Latin America/Caribbean following World Health Organization geographic classifications (United Nations 2012). Using our base-case assumption, we calculated total IQ loss and corresponding LEP lost for each country included in this analysis (see Supplemental Material, Table S2), which were summed within subregions and then combined to derive totals for each of the three major regions. From our calculations, we estimated reduced cognitive potentials (loss of IQ points) due to preventable childhood lead exposure equal to 98.2 million points in Africa, 283.6 million in Asia, and 24.4 million in Latin America/Caribbean, which translate into economic losses equal to $134.7, $699.9, and $142.3 billions of international dollars, respectively (Table 3). If we consider these losses in proportion to an estimated world PPP GDP of $81.2 trillion in 2011 (World Bank 2011), these amount to 1.20% of the global GDP.

**Table 3:** LEP lost in Africa, Asia, Latin America/Caribbean for each 1-year cohort of children < 5 years of age.

WHO region	WHO subregion	Presumed IQ loss (millions of points)	Lost LEP per IQ point (base case)	Population in each 1-year cohort of < 5 years (million, except Caribbean)	Lost LEP per 1-year cohort of <5 years [billions of international dollars (range, including both sensitivity analyses)]
Africa	Northern Africa	15.3	$26,400	4.7	$48.4 ($41.1–$57.6)
Africa	Eastern Africa	36.0	$16,500	10.6	$23.1 ($20.4–$29.1)
Africa	Western Africa	28.9	$13,100	10.2	$27.9 ($24.6–$51.6)
Africa	Middle Africa	14.7	$17,600	4.4	$14.9 ($13.1–$17.8)
Africa	Southern Africa	3.8	$20,500	1.2	$20.3 ($17.8–$24.0)
Africa	Total	98.6	$94,100	31.1	$134.7 ($118.5–$160.3) or 4.03% of GDP PPP (3.54%–4.80%)
Asia	Eastern Asia	55.1	$6,300	16.4	$227.7 ($193.9–$270.9)
Asia	Southern Asia	176.3	$17,600	36.3	$325.1 ($238.7–$386.9)
Asia	Southeastern Asia	36.4	$23,100	10.7	$90.2 ($76.8–$107.4)
Asia	Western Asia	10.9	$36,500	4.1	$42.2 ($22.5–$50.2)
Asia	Central Asia	4.9	$15,200	1.3	$14.7 ($12.9–$17.5)
Asia	Total	283.6	$98,600	68.8	$699.9 ($532.0–$832.9) or 1.88% of GDP PPP (1.43%–2.24%)
Latin America/Caribbean	Central America	7.1	$35,200	3.3	$42.0 ($18.2–$50.0)
Latin America/Caribbean	South America	15.9	$64,000	6.8	$96.2 ($59.9– $114.5)
Latin America/Caribbean	Caribbean	1.5	$54,800	514,000	$4.1 ($3.6– $4.9)
Latin America/Caribbean	Total	24.5	$154,000	10.6	$142.3 ($78.1–$169.3) or 2.04% of GDP PPP (1.12%–2.42%)
WHO, World Health Organization. Economic losses for all countries in Middle Africa, Central Asia, and for the Caribbean could be calculated only using our regression model to estimate country BLL, because no recent or complete data were available for which to use the method described by Fewtrell et al. (2003)					

In Africa the highest estimated total losses in economic productivity are in Northern and Western Africa (Table 3). Egypt and South Africa are the countries with the largest costs, with estimated losses equal to $17.8 and $17.7 billion, respectively (see Supplemental Material, Table S2). Of note, these economic losses correspond to 4.03% of African PPP GDP (Table 3). In Asia, Eastern and Southern Asia account for most of the lost economic productivity in the continent. China, with estimated losses equal to $227.1 billion, and India, with losses equal to $236.1 billion, shoulder the largest proportion of these costs. South America accounts for most of the economic losses in Latin America/Caribbean: Brazil bears the largest burden, with losses estimated at $33.1 billion.

*Sensitivity analysis.* Our sensitivity analyses suggest a range of economic losses in the range of $118.5–$160.3 billion in Africa, $78.1–$169.3 billion in Latin America/Caribbean, and $532.0–$832.9 billion in Asia (Table 3). Globally, our sensitivity analysis produces a range of $728.6–$1162.5 billion (0.90–1.43% of global GDP).

## Discussion

The principal finding of our analysis is that, despite a decline in blood lead concentration worldwide, lead exposure still represents a major contributor to children’s intellectual disability in many LMICs. This, in turn, translates into significant earning losses over a lifetime, which we estimated at 1.20% of the world GDP. Economic losses due to lead exposures in children will continue unless measures to prevent lead exposure are implemented in all countries.

For our estimates, we focused on loss of IQ and its impact on earning potential, which has been the subject of several analyses (e.g., Grosse et al. 2002). In general, the impact of IQ on earnings can be considered the result of direct effects, such as lower cognitive capacities, and indirect effects due to diminished educational achievements and reduced labor force participation. From a population perspective, even a small loss in IQ score has important repercussions on losses of potential earnings. Indeed, although an apparently small change of, for example, a 1-point decrease in IQ score might not be significant at the individual level, at the population level this will shift the distribution of IQ and increase the number of individuals who are below the normal range (Bellinger 2004).

Estimating and aggregating future earnings foregone, or lost LEP, provide a sense of the potential economic benefits of preventing childhood lead exposure that persists in LMICs. We consider our estimates to be conservative because we did not take into account consequences of lead exposure later in life, such as cardiovascular consequences. Furthermore, in our analysis, we excluded data regarding blood lead concentration near hot spots, probably underestimating the burden of intellectual disability and therefore the associated economic losses. We did not include other societal costs that may result from childhood lead exposure, such as violence and antisocial behaviors. Previous work suggests that cardiovascular disease, violence, and other related costs may be equivalent to or greater than the lost economic productivity costs described here (Gould 2009; Nevin et al. 2008).

Following the phaseout of leaded gasoline in most countries, mean BLLs have significantly declined around the world (UNEP 2012), with an estimated global benefit of US$2.45 trillion/year, 4% of world GDP in 2008 (Tsai and Hatfield 2011). However, it is now increasingly recognized that lead affects cognitive and behavioral development at levels lower than previously thought (Grandjean 2010), so full benefits of preventing childhood lead exposure have yet to be realized. In many areas of the world included in our analysis, BLLs are still significantly elevated, well above the new levels currently established by the U.S. Centers for Disease Control and Prevention (2012). Although the disease burden attributable to lead has been estimated at a global level (Fewtrell et al. 2004), economic evaluations to estimate the costs of this burden, especially lost LEP due to childhood lead exposure, have been conducted mainly in the United States and in Europe (Gould 2009; Pichery et al. 2011), but not in most developing countries, which currently stand to lose the most from this hazardous chemical exposure. Perhaps in contrast to 30 years ago, when lead poisoning was best documented in the industrialized world, a disproportionate burden of lead-associated disability and economic cost is now borne by developing countries. For comparison, U.S. and Europe lead-attributable economic costs have been estimated at $50.9 and $55 billion, respectively (Bartlett and Trasande 2013; Trasande and Liu 2011), compared with $977 billion in LMICs, suggesting that this is where most of the losses are nowadays.

Although we applied data from an international pooled analysis to assess BLL–IQ point relationships (Lanphear et al. 2005), this relationship is based on the results of studies done largely in high-income countries, assuming that the relationship is similar among children from LMICs. This might be true, but given the different comorbidities among children in high-income versus LMIC countries, we cannot be certain that such relationship apply in these countries as well.

We applied U.S. data relating IQ to percent economic productivity to estimate lost productivity across LMIC countries. As Salkever (1995) points out, technological change associated with economic growth increases the impact of IQ on productivity. In 1995, the United States had already achieved dramatic technological growth. If LMIC technological growth is greater than in the United States, then the impact of IQ (and lead exposure) on productivity in LMICs is greater than we estimated, and we are therefore likely to have underestimated the lost economic productivity due to childhood lead poisoning. We also appreciate that there is significant variability in technological growth rates across the LMICs, so there is potential for great uncertainty introduced by using an input from a single, industrialized nation.

We also extrapolated individual-level lifetime economic productivity from U.S. data, applying a PPP GDP correction factor. Given the much higher rates of growth in GDP per capita in LMICs such as China, India, and several Southeast Asian countries, annual productivity gains are almost certainly higher than in the United States, so we have likely underestimated the losses for these countries. Extrapolation from U.S. data, however, once again produces uncertainty in the estimates produced for childhood lead poisoning costs at the country level, given that GDP growth varies so widely among LMICs, leading to overestimation for some countries as well as underestimation for others.

We also assume that each country has a similar rate of decline in BLL in relationship to the year in which leaded gasoline use was phased out. Another limitation on our analysis is that for some regions of the world, very little information was available, highlighting the need to collect more data on BLLs. However, using our regression model to predict BLLs in countries for which data were not available represented a significant advantage over the method used by Fewtrell et al. (2004), allowing us to estimate BLLs in regions of the world (Middle Africa, Central Asia, and Latin America/Caribbean) for which recent or complete data were not available.

We acknowledge that the literature examining the effects of multiple chemical exposures and their potential synergistic interactions is still not well developed, and that any quantification of the economic consequences of lead exposure is inherently limited by the available data. Measurements of BLLs were performed in different laboratories, and not all of them with adequate accreditation or standard reference material, significantly increasing variability and the possibility of measurement errors.

Although a comprehensive quantification of the potential economic benefits of preventing lead exposures is still at an early stage, there is evidence suggesting that in the United States, the net benefits of lead hazard control total US$181–$269 billion, whereas the estimated costs range from $1.2 to $11.0 billion (Gould 2009). Most payers consider only the upfront expenses required for lead hazard control, because most of the benefits will occur in the future; therefore, there is no immediate return on the investment. However, the long-term returns are great: A more recent analysis conducted in the United States suggests that the estimated benefits deriving from treatment of residential lead-based paint hazards are 2–20 times higher than the estimated costs of remediation (Jones 2012). In one of the few studies conducted in the developing world, Ogunseitan and Smith (2007) estimated that lead exposure accounts for 7–25% of the disease burden among Nigerian children, and a 50% decrease in childhood BLL could save $1 billion per year. In addition, a lead abatement program lowering national BLL to 1 µg/dL by 2020 would realize a saving in the range of $2.7–8.0 billion.

Persistence of lead in the environment, in addition to uncontrolled release, will contribute to population burden for a long time to come if interventions are not initiated now. Because there is no “safe” blood lead threshold for children, and medical treatments are of limited value, the only way to avoid the large economic costs related to lead exposure is primary prevention. Also, the cost of medical treatment, if provided, might be higher than interventions aimed at preventing exposure (at least in some countries) and does not reverse the damage (Grosse et al. 2002).

## Conclusions

Our estimates suggest that lost LEP associated with childhood lead exposure in LMICs currently amounts to $977 billion annually. These findings are consistent with those of other studies and confirm that a large economic burden could be avoided if policy interventions to prevent lead exposures are implemented. Any costs of lead control interventions are at least partly offset (if not entirely offset, in some circumstances) by the health and economic benefits deriving from reducing exposure; this is a strong argument to continue to investing in lead hazard control. Nongovernmental and governmental funding entities should consider the cost of global lead poisoning as an area of ongoing concern: Without adequate preventive measures, the cost of inaction is represented by substantial economic losses and health consequences for society as a whole for generations to come.

## Supplemental Material

(213 KB) PDF
