# Supplementary material for: Economic Costs of Childhood Lead Exposure in Low- and Middle-Income Countries
Source: Environ Health Perspect. 2013 Jun 25;121(9):1097–102. doi: 10.1289/ehp.1206424 (PMC3764081; doi:10.1289/ehp.1206424)
Supplement: (213 KB) PDF [file ehp.1206424.s001.pdf]

SUPPLEMENTAL MATERIAL

Economic Costs of Childhood Lead Exposure in Low- and Middle-Income Countries

Teresa M. Attina and Leonardo Trasande

Corresponding Author: Teresa M. Attina, Department of Pediatrics, New York University School of Medicine, 227 East 30th Street Rm 703J, New York, NY 10016, teresa.attina@gmail.com, phone 646-501-2775, fax 212-263-4053.

Table of contents

Methods……………………………………………………………………………….3

Table S1………………………………………………………………………………4

Table S2………………………………………………………………………………7

References……………………………………………………………………………10

Methods

Calculation of weighted means and standard deviations

For countries in which more than one study reporting blood lead concentration was available, after estimating BLL in 2008 using our regression model, we combined these estimates to derive a single, sample-size-weighted geometric mean, according to the method previously described by Fewtrell (Fewtrell et al. 2003; Fewtrell et al. 2004).

First, we weighted the data for sample size, by transforming BLL reported in each study into the natural logarithm and then multiplying by the sample size. These values were summed and divided by the total sample size, and from the natural antilogarithm of this average we then obtained the weighted BLL mean. With respect to standard deviations from different studies, these were combined using the same method but calculated as the variance (Fewtrell et al. 2003; Fewtrell et al. 2004). Below is a working example with BLL from Argentina.

Weighted Mean

Country	Sample size (N)	Mean BLL (A)	ln(A)	ln(A) * N
Argentina	74	3.3	1.19	88.35
Argentina	203	3.2	1.16	236.12
SUM	277			324.47
Log mean	1.17			
**Weighted mean**	**3.23**			

Weighted Standard Deviation

Country	Sample size (N)	SD	SD^2	$\ln(SD^2)$	$\ln(SD^2)*N$
Argentina	74	2.1	4.41	1.48	109.81
Argentina	203	1.56	2.4336	0.89	180.54
SUM	277				290.35
$N/\ln(SD^2)*N$	1.05				
Square root	1.02				
**Weighted SD**	**2.78**				

Table S1. Sources of Blood Lead Data

Country	Subegion	Year of study	Mean BLL [µg/dl]	Standard Deviation [µg/dl]	Sample Size	Sex	Age or age group	Author source
Kenya	Eastern Africa	2008	5.99	2.42	387	Both	6-59months	Olewe et al
Kenya	Eastern Africa	2008	5.9	4.9	120	Both	≤20yrs	Njoroge et al
Uganda	Eastern Africa	2010	7.15	5.29	163	Both	4-8yrs	Graber et al
Egypt	Northern Africa	2000	22.13	8.56	30	Both	6-12yrs	Omar et al
Egypt	Northern Africa	2000	14.2	3.65	30	Both	6-12yrs	Omar et al
Egypt	Northern Africa	2002	4.81	2.97	79	Both	3-15yrs	Sharaf et al
Morocco	Northern Africa	2008	7.1		39	Both	7-14yrs	Hruba et al
Botswana	Southern Africa	2007	8.8	5.6	213	Both	1-6yrs	Mbongwe et al
South Africa	Southern Africa	2007	7.9	4.3	1581	Both	5-12yrs	Mathee et al
South Africa	Southern Africa	2003	5.7		1546	Both	13yrs	Naicker et al
South Africa	Southern Africa	2002	6.4		429	Both	7yrs	Mathee et al
South Africa	Southern Africa	2003	5.5	2.33	98	Both	5-12yrs	Norman et al
South Africa	Southern Africa	2000	7.5	3.31	1166	Both	0-4yrs	Norman et al
South Africa	Southern Africa	2000	13.2	3.5	68	Both	6-10yrs	Von Schirnding et al
Nigeria	Western Africa	2007	9.4	4.2	306	Both	1-6yrs	Ogunseitan et al
Nigeria	Western Africa	2007	8.9	4.8	653	Both	2-9yrs	Nriagu et al
Nigeria	Western Africa	2004	11.2		64	Both	0-5yrs	Wright et al
China	Eastern Asia	2008	8.07		94,778	Both	0-14yrs	He et al
China	Eastern Asia	2005	6.35	2.66	1575	Both	3-5yrs	Liu et al
China	Eastern Asia	2004	6.38		938	Both	0-7yrs	Jiang et al
China	Eastern Asia	2006	4.67		44,045	Both	0-6yrs	Zhang et al
China	Eastern Asia	2007	6.4	2.6	846	Both	10-18yrs	Zhou et al
China	Eastern Asia	2001	9.68		2,262	Both	0-6	Chen et al
China	Eastern Asia	2007	7.12	4.68	61	Both	<15yrs	Lin et al
Bangladesh	Southern Asia	2000	15		779	Both	4-12yrs	Kaiser et al
Bangladesh	Southern Asia	2008	2.47	3.32	57	Both	6 months to 12 yrs	Mitra et al
Bangladesh	Southern Asia	2008	7.24	6.31	183	Both	6 months to 12 yrs	Mitra et al
Bangladesh	Southern Asia	2009	12.13		919	Both	7 months to 12 yrs	Mitra et al
India	Southern Asia	2009	11.8		195	Both	9-14yrs	Srinivasa et al
India	Southern Asia	2006	9.32	6.21	200	Both	3-12yrs	Ahamed et al
India	Southern Asia	2003	8.36	5.23	754	Both	<12yrs	Nichani et al
India	Southern Asia	2006	11.47	5.33	756	Both	3-7yrs	Roy et al
India	Southern Asia	2009	7.47		100	Both	3-5yrs	Chaudhary et al

Country	Subegion	Year of study	Mean BLL [µg/dl]	Standard Deviation [µg/dl]	Sample Size	Sex	Age or age group	Author source
Pakistan	Southern Asia	2000	15.6		400	Both	36-60months	Rahbar et al
Pakistan	Southern Asia	2000	16.08	6.29	138	Both	6-10yrs	Rahman et al
Pakistan	Southern Asia	2000	6.48	2.71	123	Both	1-6yrs	Khan et al
Sri Lanka	Southern Asia	2003	4.33	1.81	40	Both	1-15yrs	Senanayake et al
Indonesia	South-Eastern Asia	2001	8.6		423	Both	6-12yrs	Albalak et al
Philippines	South-Eastern Asia	2004	6.9	7.7	2861	Both	6-59months	Riddell et al
Thailand	South-Eastern Asia	2010	7.71	4.62	213	Both	3-7 yrs	Neesanan et al
Thailand	South-Eastern Asia	2004	5.65	3.05	296	Both		Chomchai et al
Jordan	Western Asia	2000	3.22		382	Both	2-6yrs	Safi et al
Occupied Palestinian Territory	Western Asia	2000	4.2	3.36	344	Both	2-6yrs	Safi et al
Turkey	Western Asia	2002	3.56	1.7	180	Both	1-17yrs	Kirel et al
Turkey	Western Asia	2000	8.4		760	Both	11-13yrs	Ozden et al
Turkey	Western Asia	2001	3.67	3.88	587	Both	2-16yrs	Kismet et al
Yemen	Western Asia	2008	3.68	2.57	60	Both	6mo-6yrs	Alhemiary et al
Belize	Central America	2002	4.94	2.46	164	Both	2-8yrs	Charalambous et al
Mexico	Central America	2000	5	2.9	455	Both	2yrs	Claus Henn et al
Mexico	Central America	2002	6.2	1.5	34	Both	6-10yrs	Schnaas et al
Mexico	Central America	2000	4.9	1.6	100	Both	1-10yrs	Schnaas et al
Argentina	South America	2006	3.78		74	Both	6mo-5yrs	Disalvo et al
Argentina	South America	2000	5.2	0.5	203	Both	13mo-5yrs	Garcia et al
Brazil	South America	2006	6.2	4	97	Both	0-5yrs	Ferron et al
Brazil	South America	2008	1.48	1.2	48	Both	1-<11yrs	Menezes-Filho et al
Brazil	South America	2009	2.4		444	Both	6-8yrs	Costa de Almeida et al
Brazil	South America	2008	5.5	2.34	62	Both	0-16yrs	Oliveira da Costa Mattos et al
Brazil	South America	2000	3.05	1.96	200	Both	8-12yrs	Alvarez-Leite et al
Colombia	South America	2004	4.74		189	Both	5-9yrs	Olivero-Verbel et al
Colombia	South America	2010	3	1.2	194	Both	6-14yrs	Filigrana et al
Ecuador	South America	2010	3.17		69	Both	7-11yrs	Hruba et al
Peru	South America	2002	3.3	1.65	26	Both	3-12yrs	Naeher et al
Peru	South America	2010	8.4	3.7	120	Both	0-17yrs	Anticona et al
Uruguay	South America	2004	5.7	2.3	180	Both	0-15yrs	Cousillas et al
Uruguay	South America	2007	9	6	222	Both	5-45mo	Queirolo et al
Uruguay	South America	2010	5.7	2.8	88	Both	13-55mo	Kordas et al
Uruguay	South America	2003	9.4	2.9	112	Both	2-14yrs	Cousillas et al

Country	Subegion	Year of study	Mean BLL [µg/dl]	Standard Deviation [µg/dl]	Sample Size	Sex	Age or age group	Author source
Venezuela	South America	2000	11.62		243	Both	1-12yrs	Rojas et al
Jamaica	Caribbean	2005	4.35		1081	Both	2-6yrs	Lalor et al

Table S2. Total PPP GDP, lost LEP and corresponding percentage of GDP lost to lead-attributable IQ loss in Africa, Asia, and Latin America & the Caribbean.

Country	Region	Subregion	Total PPP GDP ($)	Lost LEP per each 1-year cohort of under 5yrs ($)	% of GDP lost to lead-attributable IQ loss
Burundi	Africa	Western Africa	5,214,000,000	179,785,229	3.45
Comoros	Africa	Western Africa	843,000,000	49,635,805	5.89
Djibouti	Africa	Western Africa	1,997,000,000	108,961,500	5.46
Eritrea	Africa	Western Africa	3,189,000,000	169,339,796	5.31
Ethiopia	Africa	Western Africa	94,604,000,000	4,474,799,004	4.73
Kenya	Africa	Western Africa	71,498,000,000	3,759,544,820	5.26
Madagascar	Africa	Western Africa	20,725,000,000	1,187,707,142	5.73
Malawi	Africa	Western Africa	14,124,000,000	878,020,196	6.22
Mauritius	Africa	Western Africa	18,677,000,000	402,814,536	2.16
Mozambique	Africa	Western Africa	23,499,000,000	1,354,089,047	5.76
Rwanda	Africa	Western Africa	13,691,000,000	789,725,385	5.77
Somalia	Africa	Western Africa	Not Available	370,908,871	
Uganda	Africa	Western Africa	46,730,000,000	3,541,522,830	7.58
Tanzania	Africa	Western Africa	68,218,000,000	4,135,136,684	6.06
Zambia	Africa	Western Africa	21,870,000,000	1,441,579,133	6.59
Zimbabwe	Africa	Western Africa	Not Available	297,764,292	
Botswana	Africa	Southern Africa	29,959,000,000	1,492,620,701	4.98
Lesotho	Africa	Southern Africa	3,762,000,000	167,728,418	4.46
Namibia	Africa	Southern Africa	15,863,000,000	708,437,400	4.47
South Africa	Africa	Southern Africa	558,216,000,000	17,708,263,135	3.17
Swaziland	Africa	Southern Africa	6,512,000,000	268,349,295	4.12
Benin	Africa	Western Africa	14,813,000,000	861,084,536	5.81
Burkina Faso	Africa	Western Africa	22,220,000,000	1,376,581,795	6.20
Cape Verde	Africa	Western Africa	2,064,000,000	75,357,268	3.65
Cote d'Ivoire	Africa	Western Africa	36,338,000,000	1,761,748,023	4.85
Gambia	Africa	Western Africa	3,793,000,000	150,083,277	3.96
Ghana	Africa	Western Africa	47,035,000,000	2,150,990,737	4.57
Guinea	Africa	Western Africa	11,534,000,000	670,831,259	5.82
Guinea-Bissau	Africa	Western Africa	1,936,000,000	105,574,120	5.45
Liberia	Africa	Western Africa	2,382,000,000	105,824,893	4.44
Mali	Africa	Western Africa	17,401,000,000	1,150,230,877	6.61
Mauritania	Africa	Western Africa	9,106,000,000	359,280,161	3.95
Niger	Africa	Western Africa	11,763,000,000	809,070,406	6.88
Nigeria	Africa	Western Africa	411,372,000,000	16,218,372,547	3.94
Senegal	Africa	Western Africa	25,288,000,000	1,491,856,637	5.90
Sierra Leone	Africa	Western Africa	5,260,000,000	297,503,901	5.66
Togo	Africa	Western Africa	6,414,000,000	319,422,821	4.98
Angola	Africa	Middle Africa	116,345,000,000	7,667,370,737	6.59
Cameroon	Africa	Middle Africa	47,738,000,000	2,520,424,705	5.28
Central African Republic	Africa	Middle Africa	3,661,000,000	185,048,267	5.05
Chad	Africa	Middle Africa	17,645,000,000	990,098,783	5.61
Congo	Africa	Middle Africa	18,337,000,000	990,089,075	5.40
DRC	Africa	Middle Africa	25,440,000,000	1,524,789,582	5.99
Gabon	Africa	Middle Africa	24,487,000,000	1,041,752,895	4.25
Sao Tome & Principe	Africa	Middle Africa	347,000,000	16,164,879	4.66
Algeria	Africa	Northern Africa	313,552,000,000	11,832,235,381	3.77
Egypt	Africa	Northern Africa	521,964,000,000	17,786,045,188	3.41

Country	Region	Subregion	Total PPP GDP ($)	Lost LEP per each 1-year cohort of under 5yrs ($)	% of GDP lost to lead-attributable IQ loss
Libya	Africa	Northern Africa	105,555,000,000	4,510,938,345	4.27
Morocco	Africa	Northern Africa	163,659,000,000	5,647,046,979	3.45
Sudan	Africa	Northern Africa	95,555,000,000	4,936,802,415	5.17
Tunisia	Africa	Northern Africa	100,496,000,000	3,666,261,844	3.65
China	Asia	Eastern Asia	11,379,182,000,000	227,193,796,810	2.00
Mongolia	Asia	Eastern Asia	13,340,000,000	490,771,006	3.68
Afghanistan	Asia	Southern Asia	41,328,000,000	2,740,680,604	6.63
Bangladesh	Asia	Southern Asia	269,127,000,000	15,877,470,243	5.90
Bhutan	Asia	Southern Asia	4,289,000,000	154,908,540	3.61
India	Asia	Southern Asia	4,533,919,000,000	236,075,873,647	5.21
Iran	Asia	Southern Asia	839,572,000,000	29,127,897,851	3.47
Nepal	Asia	Southern Asia	38,302,000,000	1,533,245,125	4.00
Pakistan	Asia	Southern Asia	488,420,000,000	37,849,991,695	7.75
Sri Lanka	Asia	Southern Asia	117,282,000,000	1,761,117,825	1.50
Kazakhstan	Asia	Southern Asia	218,393,000,000	8,179,244,100	3.75
Kyrgyzstan	Asia	Southern Asia	13,348,000,000	463,057,489	3.47
Tajikistan	Asia	Southern Asia	16,327,000,000	830,269,327	5.09
Turkmenistan	Asia	Southern Asia	46,889,000,000	1,468,564,391	3.13
Uzbekistan	Asia	Southern Asia	97,116,000,000	3,757,477,351	3.87
Cambodia	Asia	South-Eastern Asia	33,925,000,000	1,294,491,599	3.82
Indonesia	Asia	South-Eastern Asia	1,131,166,000,000	37,898,249,791	3.35
Lao PDR	Asia	South-Eastern Asia	17,664,000,000	719,501,628	4.07
Malaysia	Asia	South-Eastern Asia	449,876,000,000	11,820,685,513	2.63
Myanmar	Asia	South-Eastern Asia	Not Available	3,158,364,032	
Philippines	Asia	South-Eastern Asia	392,679,000,000	15,019,373,494	3.82
Thailand	Asia	South-Eastern Asia	605,019,000,000	12,522,675,327	2.07
Timor-Leste	Asia	South-Eastern Asia	1,868,000,000	73,329,137	3.93
Vietnam	Asia	South-Eastern Asia	301,728,000,000	7,718,516,082	2.56
Armenia	Asia	Western Asia	18,071,000,000	413,769,244	2.29
Azerbaijan	Asia	Western Asia	92,927,000,000	2,490,181,420	2.68
Georgia	Asia	Western Asia	24,684,000,000	427,255,280	1.73
Iraq	Asia	Western Asia	128,237,000,000	7,565,979,730	5.90
Jordan	Asia	Western Asia	37,130,000,000	466,412,076	1.26
Lebanon	Asia	Western Asia	62,650,000,000	1,588,855,198	2.54
Syria	Asia	Western Asia	107,584,000,000	5,043,120,306	4.69
Turkey	Asia	Western Asia	1,288,638,000,000	22,116,719,752	1.72
Yemen	Asia	Western Asia	58,257,000,000	2,090,616,995	3.59
Belize	Latin America &the Caribbean	Central America	2,397,000,000	51,554,488	2.15
Costa Rica	Latin America &the Caribbean	Central America	57,836,000,000	1,325,312,244	2.29
El Salvador	Latin America &the Caribbean	Central America	42,829,000,000	1,305,258,131	3.05
Guatemala	Latin America &the Caribbean	Central America	73,216,000,000	3,289,701,595	4.49
Honduras	Latin America &the Caribbean	Central America	31,530,000,000	1,202,295,126	3.81
Mexico	Latin America &the Caribbean	Central America	1,752,883,000,000	32,582,356,784	1.86
Nicaragua	Latin America &the Caribbean	Central America	17,263,000,000	623,283,948	3.61

Country	Region	Subregion	Total PPP GDP ($)	Lost LEP per each 1-year cohort of under 5yrs ($)	% of GDP lost to lead-attributable IQ loss
Panama	Latin America &the Caribbean	Central America	56,051,000,000	1,607,463,745	2.87
Argentina	Latin America &the Caribbean	South America	720,488,000,000	9,781,430,507	1.36
Bolivia	Latin America &the Caribbean	South America	51,751,000,000	1,699,222,188	3.28
Brazil	Latin America &the Caribbean	South America	2,304,646,000,000	33,024,539,420	1.43
Chile	Latin America &the Caribbean	South America	298,947,000,000	6,446,169,908	2.16
Colombia	Latin America &the Caribbean	South America	474,113,000,000	8,906,102,580	1.88
Ecuador	Latin America &the Caribbean	South America	124,462,000,000	2,458,673,422	1.98
Guyana	Latin America &the Caribbean	South America	2,599,000,000	72,269,649	2.78
Paraguay	Latin America &the Caribbean	South America	35,590,000,000	1,261,327,670	3.54
Peru	Latin America &the Caribbean	South America	303,342,000,000	10,724,435,709	3.54
Suriname	Latin America &the Caribbean	South America	4,140,000,000	123,286,437	2.98
Uruguay	Latin America &the Caribbean	South America	51,140,000,000	1,339,509,882	2.62
Venezuela	Latin America &the Caribbean	South America	375,815,000,000	20,352,716,902	5.42
Dominican Republic	Latin America &the Caribbean	Caribbean	99,186,000,000	3,154,090,570	3.18
Grenada	Latin America &the Caribbean	Caribbean	1,173,000,000	33,813,602	2.88
Haiti	Latin America &the Caribbean	Caribbean	11,940,000,000	439,851,115	3.68
Jamaica	Latin America &the Caribbean	Caribbean	21,851,000,000	387,445,439	1.77
Saint Lucia	Latin America &the Caribbean	Caribbean	1,652,000,000	53,483,894	3.24
Saint Vincent	Latin America &the Caribbean	Caribbean	1,182,000,000	32,797,994	2.77

References

Ahamed M, Verma S, Kumar A, Siddiqui MK. 2010. Blood lead levels in children of Lucknow, India. Environ Toxicol 25:48-54.

Albalak R, Noonan G, Buchanan S, Flanders WD, Gotway-Crawford C, Kim D, et al. 2003. Blood lead levels and risk factors for lead poisoning among children in Jakarta, Indonesia. Sci Total Environ 301:75-85.

Alhemiary N, Al-Duais M, Mutair A, Wassel A, Alshrabi B, Albadany B. 2011. Anodic stripping voltammetry determination of Pb, Cd, Zn, and Cu in blood samples of children in some areas of Ibb governorate. J Iran Chem Res 4:9-16.

Anticona C, Bergdahl IA, San Sebastian M. 2012. Lead exposure among children from native communities of the Peruvian Amazon basin. Rev Panam Salud Publica 31:296-302.

Charalambous A, Demoliou K, Mendez M, Coye R, Solorzano G, Papanastasiou E. 2009. Screening for lead exposure in children in Belize. Rev Panam Salud Publica 25:47-50.

Chaudhary V, Sharma MK. 2011. Blood lead level in the children of Western Uttar Pradesh, India. Toxicol Environ Chem 93:504-512

Chen XX, Teng HH, Wang FZ, He JP, Zhou SZ, Jian YJ, et al. 2003. [Blood lead level and related risk factors among children aged 0-6 years in Beijing]. Zhonghua Liu Xing Bing Xue Za Zhi 24:868-871.

Chomchai C, Padungtod C, Chomchai S. 2005. Predictors of elevated blood lead level in Thai children: a pilot study using risk assessment questionnaire. J Med Assoc Thai 88 Suppl 8:S53-59.

Claus Henn B, Schnaas L, Ettinger AS, Schwartz J, Lamadrid-Figueroa H, Hernandez-Avila M, et al. 2012. Associations of early childhood manganese and lead coexposure with neurodevelopment. Environ Health Perspect 120:126-131.

Costa de Almeida GR, de Sousa Guerra C, de Angelo Souza Leite G, Antonio RC, Barbosa F, Jr., Tanus-Santos JE, et al. 2011. Lead contents in the surface enamel of primary and permanent teeth, whole blood, serum, and saliva of 6- to 8-year-old children. Sci Total Environ 409:1799-1805.

Cousillas AZ, Manay N, Pereira L, Alvarez C, Coppes Z. 2005. Evaluation of lead exposure in Uruguayan children. Bull Environ Contam Toxicol 75:629-636

Cousillas AZ, Pereira L, Alvarez C, Heller T, De Mattos B, Piastra C, et al. 2008. Comparative study of blood lead levels in Uruguayan children (1994–2004). Biol Trace Elem Res 122:19-25.

Disalvo L, Aab C, Pereyras S, Pattin J, Apezteguia M, Iannicelli JC, et al. 2009. [Blood lead levels in children from the city of La Plata, Argentina. Relationship with iron deficiency and lead exposure risk factors]. Arch Argent Pediatr 107:300-306.

Ferron MM, Lima AK, Saldiva PH, Gouveia N. 2012. Environmental lead poisoning among children in Porto Alegre state, Southern Brazil. Rev Saude Publica 46:226-233.

Fewtrell L, Kaufman RB, Pruss-Ustun A. 2003. Lead: Assessing the environmental burden of disease at national and local levels. Geneva, world health organization. Available at http://www.Who.Int/quantifying_ehimpacts/publications/9241546107/en/index.Html (accessed May 1, 2012).

Fewtrell LJ, Pruss-Ustun A, Landrigan P, Ayuso-Mateos JL. 2004. Estimating the global burden of disease of mild mental retardation and cardiovascular diseases from environmental lead exposure. Environmental Research 94(2):120-133.

Filigrana PA, Mendez F. 2012. Blood lead levels in schoolchildren living near an industrial zone in Cali, Colombia: the role of socioeconomic condition. Biol Trace Elem Res 149:299-306.

Garcia SI, Mercer R. 2003. [Childhood health and lead in Argentina]. Salud Publica Mex 45 Suppl 2:S252-255.

Graber LK, Asher D, Anandaraja N, Bopp RF, Merrill K, Cullen MR, et al. 2010. Childhood lead exposure after the phaseout of leaded gasoline: an ecological study of school-age children in Kampala, Uganda. Environ Health Perspect 118:884-889.

He K, Wang S, Zhang J. 2009. Blood lead levels of children and its trend in China. Sci Total Environ 407:3986-3993.

Hruba F, Stromberg U, Cerna M, Chen C, Harari F, Harari R, et al. 2012. Blood cadmium, mercury, and lead in children: an international comparison of cities in six European countries, and China, Ecuador, and Morocco. Environ Int 41C:29-34.

Jiang YM, Shi H, Li JY, Shen C, Liu JH, Yang H. Environmental lead exposure among children in Chengdu, China: blood lead levels and major sources. Bull Environ Contam Toxicol 84:1-4.

Kaiser R, Henderson AK, Daley WR, Naughton M, Khan MH, Rahman M, et al. 2001. Blood lead levels of primary school children in Dhaka, Bangladesh. Environ Health Perspect 109:563-566.

Khan DA, Ansari WM, Khan FA. 2011. Synergistic effects of iron deficiency and lead exposure on blood lead levels in children. World J Pediatr 7:150-154.

Kirel B, Aksit MA, Bulut H. 2005. Blood lead levels of maternal-cord pairs, children and adults who live in a central urban area in Turkey. Turk J Pediatr 47:125-131.

Kismet E, Karatas M, Demirkaya E, Atay A, Unay B, Aydin A, et al. 2004. [Blood lead levels in children residing in different locations in Ankara]. Gulhane Med J 46:033-037.

Kordas K, Ardoino G, Ciccariello D, Manay N, Ettinger AS, Cook CA, et al. 2011. Association of maternal and child blood lead and hemoglobin levels with maternal perceptions of parenting their young children. Neurotoxicology 32:693-701.

Lalor G, Vutchkov M, Bryan S. 2007. Blood lead levels of Jamaican children island-wide. Sci Total Environ 374:235-241.

Lin S, Wang X, Yu IT, Tang W, Miao J, Li J, et al. 2011. Environmental lead pollution and elevated blood lead levels among children in a rural area of China. Am J Public Health 101:834-841.

Liu J, McCauley LA, Zhao Y, Zhang H, Pinto-Martin J. 2009. Cohort profile: The China Jintan child cohort study. Int J Epidemiol 39:668-674.

Mathee A, Naicker N, Barnes B. 2009. Blood lead levels in South African children at the end of the leaded petrol era. Preliminary abridged report: South African Medical Research Council.

Mathee A, von Schirnding YE, Levin J, Ismail A, Huntley R, Cantrell A. 2002. A survey of blood lead levels among young Johannesburg school children. Environ Res 90:181-184.

Mbongwe B, Barnes B, Tshabang J, Tsai M, Rajoram S, Mpuchane S, et al. 2010. Exposure to lead among children aged 1-6 years in the city of Gaborone, Botswana. J Environ Health Res 10:17-26.

Menezes-Filho JA, Viana GF, Paes CR. 2012. Determinants of lead exposure in children on the outskirts of Salvador, Brazil. Environ Monit Assess 184:2593-2603.

Mitra AK, Haque A, Islam M, Bashar SA. 2009. Lead poisoning: an alarming public health problem in Bangladesh. Int J Environ Res Public Health 6:84-95.

Mitra AK, Saha PK. Lead poisoning in young children in Bangladesh: need for educational intervention. In: Proceedings of the 13th Annual Scientific Conference (ASCON). ICDDR, B. Dhaka, Bangladesh. Available at http://wwwicddrborg/what-we-do/publications/cat_view/52-publications/10042-icddrb-periodicals/10073-scientific-conferences/10081-13th-ascon-2011 (Accessed March 15 2012), 2011.

Naeher LP, Aguilar-Villalobos M, Miller T. 2004. Blood lead survey of children, pregnant women, professional drivers, street workers, and office workers in Trujillo, Peru. Arch Environ Health 59:359-362.

Naicker N, Norris SA, Mathee A, von Schirnding YE, Richter L. 2010. Prenatal and adolescent blood lead levels in South Africa: child, maternal and household risk factors in the birth to twenty cohort. Environ Res 110:355-362.

Neesanan N, Kasemsup R, Ratanachuaeg S, Kojaranjit P, Sakulnoom K, Padungtod C. 2011. Preliminary study on assessment of lead exposure in Thai children aged between 3-7 years old who live in Umphang district, Tak province. J Med Assoc Thai 94 Suppl 3:S113-12.

Nichani V, Li WI, Smith MA, Noonan G, Kulkarni M, Kodavor M, et al. 2006. Blood lead levels in children after phase-out of leaded gasoline in Bombay, India. Sci Total Environ 363:95-106.

Njoroge GK. 2005. Blood lead levels in Kenya: A case study for children and adolescents in selected areas of Nairobi and Olkalou, Nyandarua district. In cooperation with the United Nations Environment Programme (UNEP).

Norman R, Bradshaw D, Lewin S, Cairncross E, Nannan N, Vos T. 2010. Estimating the burden of disease attributable to four selected environmental risk factors in South Africa. Rev Environ Health 25:87-119.

Norman R, Mathee A, Barnes B, van der Merwe L, Bradshaw D. 2007. Estimating the burden of disease attributable to lead exposure in South Africa in 2000. S Afr Med J 97:773-780.

Nriagu J, Afeiche M, Linder A, Arowolo T, Ana G, Sridhar MK, et al. 2008. Lead poisoning associated with malaria in children of urban areas of Nigeria. Int J Hyg Environ Health 211:591-605.

Ogunseitan OA, Smith TR. 2007. The cost of environmental lead poisoning (Pb) in Nigeria. African Journal of Environmental Science and Technology 1:27-36.

Olewe TM, Mwanthi MA, Wang'ombe JK, Griffiths JK. 2009. Blood lead levels and potential environmental exposures among children under five years in Kibera slums, Nairobi. East Afr J Public Health 6:6-10.

Oliveira da Costa Mattos Rde C, Xavier EC, Jr., Domingos Mainenti HR, Mitri Nogueira S, Ribeiro de Carvalho MA, Ramos Moreira Mde F, et al. 2009. Evaluation of calcium excretion in Brazilian infantile and young population environmentally exposed to lead. Hum Exp Toxicol 28:567-575.

Olivero-Verbel J, Duarte D, Echenique M, Guette J, Johnson-Restrepo B, Parsons PJ. 2007. Blood lead levels in children aged 5-9 years living in Cartagena, Colombia. Sci Total Environ 372:707-716.

Omar M, Ibrahim M, Assem H, Moustafa Y, Battah F. 2001. Teeth and blood lead levels in Egyptian schoolchildren: relationship to health effects. J Appl Toxicol 21:349-352.

Ozden TA, Killic A, Vehida HE, Toparlakb D, Gokcay G, Sanera G. 2004. Blood lead levels in school children. Indoor Built Environ 13:149-154.

Queirolo EI, Ettinger AS, Stoltzfus RJ, Kordas K. 2010. Association of anemia, child and family characteristics with elevated blood lead concentrations in preschool children from Montevideo, Uruguay. Arch Environ Occup Health 65:94-100.

Rahbar MH, White F, Agboatwalla M, Hozhabri S, Luby S. 2002. Factors associated with elevated blood lead concentrations in children in Karachi, Pakistan. Bull World Health Organ 80:769-775.

Rahman A, Maqbool E, Zuberi HS. 2002. Lead-associated deficits in stature, mental ability and behaviour in children in Karachi. Ann Trop Paediatr 22:301-311.

Riddell TJ, Solon O, Quimbo SA, Tan CM, Butrick E, Peabody JW. 2007. Elevated blood-lead levels among children living in the rural Philippines. Bull World Health Organ 85:674-680.

Rojas M, Espinosa C, Seijas D. 2003. [Association between blood lead and sociodemographic parameters among children]. Rev Saude Publica 37:503-509.

Roy A, Hu H, Bellinger DC, Palaniapan K, Wright RO, Schwartz J, et al. 2009. Predictors of blood lead in children in Chennai, India (2005-2006). Int J Occup Environ Health 15:351-359.

Safi J, Fischbein A, El Haj S, Sansour R, Jaghabir M, Hashish M, et al. 2006. Childhood lead exposure in the Palestinian Authority, Israel, and Jordan: results from the Middle Eastern regional cooperation project, 1996-2000. Environ Health Perspect 114:917-92

Schnaas L, Rothenberg SJ, Flores MF, Martinez S, Hernandez C, Osorio E, et al. 2004. Blood lead secular trend in a cohort of children in Mexico City (1987-2002). Environ Health Perspect 112:1110-1115.

Senanayake MP, Rodrigo MD, Malkanthi R. 2004. Blood lead levels of children before and after introduction of unleaded petrol. Ceylon Med J 49:60-61.

Sharaf NE, Abdel-Shakour A, Amer NM, Abou-Donia MA, Khatab N. 2008. Evaluation of children's blood lead level in Cairo, Egypt. American-Eurasian J Agric & Environ Sci 3:414-419.

Srinivasa Reddy Y, Pullakhandam R, Radha Krishna KV, Uday Kumar P, Dinesh Kumar B. 2011. Lead and essential trace element levels in school children: a cross-sectional study. Ann Hum Biol 38:372-377.

von Schirnding Y, Mathee A, Kibel M, Robertson P, Strauss N, Blignaut R. 2003. A study of pediatric blood lead levels in a lead mining area in South Africa. Environ Res 93:259-263.

Wright NJ, Thacher TD, Pfitzner MA, Fischer PR, Pettifor JM. 2005. Causes of lead toxicity in a Nigerian city. Arch Dis Child 90:262-266.

Zhang SM, Dai YH, Xie XH, Fan ZY, Tan ZW, Zhang YF. 2009. Surveillance of childhood blood lead levels in 14 cities of China in 2004-2006. Biomed Environ Sci 22:288-296.

Zhou W, Jiang Y, Shi H, Liu J, Shen C, Dai Q, et al. An epidemiological survey of blood lead level in Tibetan youth 10-18 years old in Songpan, China. Biol Trace Elem Res 137:49-54.
